# Diversifying the concept of model organisms in the age of -omics

**DOI:** 10.1038/s42003-023-05458-x

**Published:** 2023-10-19

**Authors:** Fabrice Bertile, Sabine Matallana-Surget, Andreas Tholey, Susana Cristobal, Jean Armengaud

**Affiliations:** 1 Université de Strasbourg, CNRS, IPHC UMR 7178, 23 rue du Loess, 67037 Strasbourg Cedex 2, France; 2 Division of Biological and Environmental Sciences, Faculty of Natural Sciences, University of Stirling, Stirling, FK9 4LA UK; 3 Systematic Proteome Research & Bioanalytics, Institute for Experimental Medicine, Christian-Albrechts-Universität zu Kiel, 24105 Kiel, Germany; 4 Department of Biomedical and Clinical Sciences, Cell Biology, Medical Faculty, Linköping University, Linköping, 581 85 Sweden; 5 Ikerbasque, Basque Foundation for Science, Department of Physiology, Faculty of Medicine and Nursing, University of the Basque Country (UPV/EHU), Barrio Sarriena, s/n, Leioa, 48940 Spain; 6 Université Paris-Saclay, CEA, INRAE, Département Médicaments et Technologies pour la Santé (DMTS), SPI, 30200 Bagnols-sur-Cèze, France

**Keywords:** Philosophy, Biological models

## Abstract

In today’s post-genomic era, it is crucial to rethink the concept of model organisms. While a few historically well-established organisms, e.g. laboratory rodents, have enabled significant scientific breakthroughs, there is now a pressing need for broader inclusion. Indeed, new organisms and models, from complex microbial communities to holobionts, are essential to fully grasp the complexity of biological principles across the breadth of biodiversity. By fostering collaboration between biology, advanced molecular science and omics communities, we can collectively adopt new models, unraveling their molecular functioning, and uncovering fundamental mechanisms. This concerted effort will undoubtedly enhance human health, environmental quality, and biodiversity conservation.

## Abstract (web summary)

The concept of model organisms in biological studies needs to be re-evaluated to reflect novel technological advances and help further scientific discovery.

## Introduction

To paraphrase Krogh’s principle^1^, later reformulated by Claude Bernard^2^, the first important step in the development of a biological experimental design is the selection of relevant model organism(s). Extrapolating the knowledge gained beyond the studied specimens is indeed essential to extend our understanding to numerous species. Hence, the concept of a model organism is broad, and its choice depends on the research questions and objectives. Due to its importance in biology and significant improvement in molecular biology and genetic engineering, this key concept has been reinvestigated. As recently discussed by Ankeny and Leonelli^3^, a primary distinction should be made between experimental versus model organisms. An experimental organism is chosen to study a particular biological process and serves as a model only for its closely related species, whereas a model organism allows for the study of specific processes and its genetics and physiology, for example, can be projected onto a wider range of species, most often including humans. Therefore, the concept of model organisms is based on the principle of evolutionary conservation. Additional criteria for an organism to be considered as a suitable model generally include: i) ‘simplicity’ and universal applicability across laboratories, ii) genetic stability that ensures the production of the same organism consistently without genetic drift, iii) genomic and/or transcriptomic resources, and iv) genome-editing tools for conducting gene-loss or gene-gain experiments^4^. Consequently, a limited number of candidates meet all the above requirements and only a handful of model organisms have thus allowed major advances in life sciences^5^. For example, the roundworm *Caenorhabditis elegans*, the fruit fly *Drosophila melanogaster*, the zebrafish *Danio rerio*, and the plant *Arabidopsis thaliana* have pioneered developmental genetics^6^, the bacterium *Escherichia coli* has helped unravel the basic concepts of transcriptional regulation^7^, the budding yeast *Saccharomyces cerevisiae* has made it possible to decipher eukaryotic cell cycle and network interactions^8^, and the mouse *Mus musculus* has become the physiology and disease model of choice for humans^9^.

## Re-evaluating the concept of ‘model organism’

Despite their undeniable contribution to major discoveries in biology and medicine, well-established model organisms are not exempt from inherent limitations. The choice of simplified systems as model organisms does not always appropriately reflect the complexity of more complex systems such as the human body and its specific interactions with its long-life adapted microbiota. Indeed, animals and plants must be considered as holobionts, comprising not only their own cells, but also those of the microorganisms they host. The selection of more simplified models is at risk because of species-related specificities, which could prevent systematic extrapolation to other species. As an example of this limitation, the immunomodulator TGN1412 unexpectedly triggered a severe immune response in all six volunteers during phase I clinical studies, resulting in life-threatening multi-organ failure^10^. This occurred despite preclinical trials in various animal species concluding that the molecule was safe and effective in treating autoimmune diseases. Extrapolating results from model organisms may therefore represent a shortcut that overlooks significant differences between species. Consequently, one should approach these results with caution, as model organisms, including laboratory models, remain essential for major medical advances. Hence, the current handful of highly standardized model organisms cannot represent the complexity of all biological principles in the full breadth of biodiversity. This has long been an important claim in ecology^11^, ecotoxicology^12^, and evolutionary developmental biology^13^.

Many studies are conducted on animals in captivity and this is another possible limitation that we could identify. Not only can the confounding effects of captivity alter the healthy mental development and metabolic physiology of animal model organisms, but also an organism’s interaction with the natural environment – particularly when the environment itself undergoes changes - is not taken into account. Therefore, even individuals of well-characterized model species may differ from their counterparts that live in the wild, whether of the same or a related species^14,15^. Moreover, the use of model organisms is now recognized to have important drawbacks in answering many research questions^16^. For instance, the relevance of the mouse model - a short-lived laboratory rodent species (only 2 years of lifespan) fed only with always the same standardized food - to understand the aging processes of long-lived species may be questioned^17^, especially when alternative models like bats could be more relevant as they present significantly longer life expectancies for mammals (up to 38 years)^18^. However, studies using nematodes, flies, fish and mice retain the strong advantage of their rapid generation times and ability to facilitate rapid genetics - a challenge that is heightened when dealing with animals of considerably longer lifespans, like bats. Other models suitable for research on aging are also likely to emerge such as the killifish *Nothobranchius furzeri*^19^. This species exhibits a rapid age-dependent decline, has well-documented ecology and behavior, and is relatively easy to house and breed. These characteristics have encouraged the development of specific resources, such as high-quality genomes and transcriptomes and genetic manipulation tools. Finally, many other systems that could enable addressing several biology questions such as, e.g., the giant ciliate *Stentor coeruleus* as a model for single-cell regeneration, the filamentous fungus *Ashbya gossypii* as a model for cytoplasm organization, or eusocial insects as a model for sociality-related processes^20,21^.

Classical research approaches aim to search for models that will mimic the symptoms of a disease, hence allowing to understand the progression of a disease state. Conversely, biodiversity offers numerous alternative models that allow to determine how wildlife succeeds where humans fail; i.e. how wild animals may resist harsh environmental conditions whereas humans would not. Such an approach clearly overlooked so far, is likely to bring discoveries with great potential, such as cancer resistance mechanisms detected in naked mole rats, which are rodents not related to mice or rats, but close to porcupines and guinea pigs. These novel regulatory mechanisms do not appear to exist in mice^22^ but involve proteins known and studied for decades in cancer research. Another example is the mechanisms evolved by bears that allow them to maintain their muscle mass and strength despite inactivity during hibernation^23^, whereas the equivalent situation of physical inactivity and/or starvation in humans would lead to disuse atrophy, possibly until death^24,25^. It is also interesting to note the hyperglycemia that characterizes birds but without the adverse effects observed in type 2 diabetics^26^ or the existence of antimicrobial peptides in penguins that would help fight infections in salt-rich body fluids in humans^27^. Hence, the pantheon of already established model organisms is limited for answering important scientific questions, thus explaining the recent increased interest in other organisms as a source of new information of biological and clinical interest. The new models can be studied in the wild or in captivity. Captivity may offer more possibilities for manipulating diet or applying specific treatments, or for repeated sampling and measurement. Studies in the wild have sometimes proven to be necessary because certain mechanisms are linked only to behavioral changes that occur spontaneously under natural conditions. This is illustrated by the definition of biomarkers of the safety limit of prolonged food deprivation, which was obtained using proteomics in wild penguins as they abandoned their nest, but not in captive penguins despite having a similar metabolic state^28^. However, conducting studies on wild populations often remains challenging, primarily due to logistical and regulatory factors, including ethical approvals and specificities of certain geographical regions.

## Proteomics has the power to help rapidly increase the number of model organisms

Today, advanced molecular tools open an avenue to the extension of the set of studied model organisms^16^. The continuous increase of fully sequenced genomes undoubtedly contributes to the development of research on a growing number of species, hence improving basic knowledge in different fields, such as microbiology^29^ or molecular ecology^30^. Importantly, the manipulation of the genomes of nearly any organism has become possible with recent genome editing approaches like CRISPR/Cas9^31,32^. Moreover, the rapid advancement of genome sequencing is enabling the assembly of new genomes for an ever-increasing number of species, thus providing an opportunity to annotate these genomes, albeit not in the most accurate manner^33^. In this way, genome sequencing fosters the development of the so-called post-genomic sciences, i.e. the various omics that have come to the forefront in the past two decades^34^. In particular, proteomics has kept evolving over the years, and it has now reached a level of performance that enables analysis from a single cell^35–38^ to samples composed of complex communities, the so-called metaproteomics commonly used on microbiomes^39^. Such progress allows, e.g., to refine our perception of the biology of heterogeneous tissues and organs, to understand host-microbiota interactions and symbiosis, to characterize more complex models such as the holobiont system^40,41^, and to qualify any species system from very low amounts of biological material, thus having the potential to transform any experimental organism into a model organism^42^. In addition to focusing on proteins, which are the real workhorses of the biological systems, one of the strengths of proteomics is that this methodology does not necessarily require sequencing and annotating genomes in advance. While the results are never as good as when genome sequences have been previously obtained for the studied species using RNAseq, this positions all studied species in the starting blocks of the race for the emergence of new model organisms. This is possible thanks to the conservation of sets of sequences across species, but also the emergence of reliable de novo protein sequencing methodologies, thus increasing the coverage of the protein sequences and proteomes for nearly all organisms^43^. Finally, next-generation sequencing has become less expensive and more effective over the years for any organism^44^, as does proteomics. In turn, proteomics data can improve genome annotations and they can be combined with other omics data within the framework of proteogenomics, a highly recommended strategy for improving our information and ability to manipulate many organisms. Importantly, the proteome directly reflects the true functional level of the “omes” by identifying and quantifying enzymes, building block proteins, and the different actors in signaling pathways, thus giving a glimpse into how the biological systems function through two variables: i) the functions carried out, and ii) their abundance which provides a proxy for their activity^39^. Proteome data therefore provide very complementary information to genome and transcriptome data, which provide information on the potential of an organism without reflecting the molecular processes that drive biological systems, an aspect that is frequently overlooked in medical research. Importantly, by bringing information on the fluxes of metabolites, metabolomics is today highly complementary to proteomics.

## A plethora of novel candidate model organisms

The promotion of research in the wealth of the non-human species that have been neglected so far is strongly supported by the ‘Initiative for Model Organism Proteomes’ (iMOP), active within the Human Proteome Organization (HUPO) and European Proteomics Association (EUPA). Today, iMOP re-evaluates the concept of ‘model organism’. In particular, we consider organisms as models when they are appropriate i) for the study of biological mechanisms important to human health and disease, ii) for a better understanding of pathogenicity, pathogen reservoirs, and the emergence of resistance, and iii) to decipher the toxic effects of pollutants and exposomes on biological systems and thus define sentinels of our environment, or to be relevant to the One-Health concept^45^. The identification and characterization of novel fundamental molecular mechanisms in numerous understudied species has the potential to bring novelty and favor innovation, such as new therapeutic/preventive levers for human health. Comparative proteomics therefore has a major role to play in evolutionary biology and medicine. Proteogenomics has great potential to foster the identification of novel coding sequences, delineate their structure and regulatory elements, and characterize the function of the encoded proteins. It is essential to document their presence in various biological systems and to comprehend any evolutionary differences among them. Advanced annotation of the proteome of specific biological models, such as the human proteome, for which a wealth of information is available, remains crucial to the advancement of biology. Furthermore, environmental proteomics and metaproteomics are interesting tools to unravel the functioning of diverse ecosystems, discover novel enzymes for biotechnological applications and/or monitor the fate of pathogens in the environment, characterize their reservoirs, evaluate their spread, and understand the development of antibiotic resistance. Toxicoproteomics will analyze the effects of environmental factors and pollutants on new model organisms representative of the biosphere, which should provide basic knowledge of major interest for the preservation of human health. Finally, monitoring the quality of the environment through the follow-up of sentinel organisms calls for enhancing the efforts on ecotoxicoproteomics^12^. Hence, next-generation proteomics could soon become a driving force in making the studies of biological niche organisms a more routine approach. The molecular processes and mechanisms optimized for these niches could be essential for humans, so model organisms must be selected both from those that can be housed in the laboratory and from wild species in their natural environment.

To successfully promote the emergence of new model organisms, efforts are required to further improve databases and tools to leverage inter-organism comparison^46^. To be able to draw parallels between the proteome of various species, including humans, would greatly facilitate the understanding of processes in the light of evolution and help determine how they could be manipulated to improve health. This will require improvements in the annotation of the genomes of representative branches of the tree of life, as well as the possibility for high-throughput sequence similarity searches and functional homology assessment, orthology prediction, function estimation, and for the comparison of post-translational modifications and maturation, relative protein abundances and regulations. Here again, the most promising approaches are proteogenomics and multi-omics strategies, which allow us to decipher how the flow of genetic information, from DNA to proteins through RNA, influences the functioning of biological systems^47,48^. An important aspect to be taken into account is the increasing understanding of the proteoform concept^49^. Proteoforms describe all protein species being formed by various genetic, transcriptional, and (post)translational processes out of a single gene. The proteoforms built by these processes can have distinct functions^50^, and numerous studies on diverse model organisms have reported the effects of splicing and/or posttranslational modifications on proteome complexity and, therefore, the greatly expanded functional capacity of proteomes^51,52^. Despite this, most studies still follow the long outdated “one gene-one protein” hypothesis, which does not reflect the entire functional potential of the proteome.

## Bridging communities – towards the best practice approach

Enhancing our understanding of the biological world is of utmost importance, particularly within the context of global warming, the emergence of new pollution, novel pathogens, and the challenge of antibiotic resistance. Bioinspired research strategies should aim at improving the overall human health, quality of the environment, and biodiversity conservation. The alliance of biologists from different backgrounds with multi-omics specialists holds the promise of utilizing any living species as a unique model organism. This collaboration fosters multidisciplinary approaches to tackle challenging scientific questions effectively. To facilitate accurate interpretation of omics data and enhance our comprehension of biological systems, collaborative efforts (among scientific communities) are pivotal in aligning on revised definitions and standardized nomenclature for proteoform, protein and gene names. This will result in enriched descriptions of biological systems and molecular pathways within the realm of comparative biology - a transformative advancement that undoubtedly benefits human health.

## References

[1] Krogh A (1929). The progress of physiology. Science.

[2] Bernard, C. Introduction à l’Etude de la Médecine Expérimentale (J.B. Baillière et Fils.; 1865).

[3] Ankeny, R. A. & Leonelli, S. *Model organisms* (Cambridge University Press, 2020). A philosophical exploration of the concept of the ‘model organism’ in contemporary biology.

[4] Matthews BJ, Vosshall LB (2020). How to turn an organism into a model organism in 10 ‘easy’ steps. J. Exp. Biol..

[5] Davis RH (2004). The age of model organisms. Nat. Rev. Genet.

[6] Irion U, Nüsslein-Volhard C (2022). Developmental genetics with model organisms. Proc. Natl Acad. Sci. USA.

[7] Jacob F, Monod J (1961). Genetic regulatory mechanisms in the synthesis of proteins. J. Mol. Biol..

[8] Sinclair D, Mills K, Guarente L (1998). Aging in *Saccharomyces cerevisiae*. Annu Rev. Microbiol.

[9] Phifer-Rixey M, Nachman MW (2015). The natural history of model organisms: insights into mammalian biology from the wild house mouse Mus musculus. eLife.

[10] Hunter P (2008). The paradox of model organisms. The use of model organisms in research will continue despite their shortcomings. EMBO Rep..

[11] Shine R, Bonnet X (2000). Snakes: a new ‘model organism’ in ecological research?. Trends Ecol. Evol..

[12] Gouveia D (2019). Ecotoxicoproteomics: a decade of progress in our understanding of anthropogenic impact on the environment. J. Proteom..

[13] Jenner RA, Wills MA (2007). The choice of model organisms in evo-devo. Nat. Rev. Genet.

[14] Alfred J, Baldwin IT (2015). The natural history of model organisms: new opportunities at the wild frontier. eLIFE.

[15] Markow TA (2015). The natural history of model organisms: the secret lives of Drosophila flies. eLife.

[16] Goldstein B, King N (2016). The future of cell biology: emerging model organisms. Trends Cell Biol..

[17] Austad SN (2009). Comparative biology of aging. J. Gerontol. A Biol. Sci. Med. Sci..

[18] Wilinson GS, South JM (2002). Life history, ecology and longevity in bats. Aging Cell.

[19] Holtze S (2021). Alternative animal models of aging research. Front. Mol. Biosci..

[20] Russell JJ (2017). Non-model model organisms. BMC Biol..

[21] Marx V (2021). Model organisms on roads less traveled. Nat. Methods.

[22] Seluanov A (2009). Hypersensitivity to contact inhibition provides a clue to cancer resistance of naked mole-rat. Proc. Natl Acad. Sci. USA.

[23] Bertile F, Habold C, Le Maho Y, Giroud S (2021). Body protein sparing in hibernators: a source for biomedical innovation. Front. Physiol..

[24] Le Maho Y (2002). Nature and function. Nature.

[25] Rudrappa SS (2016). Human skeletal muscle disuse atrophy: effects on muscle protein synthesis, breakdown, and insulin resistance-a qualitative review. Front. Physiol..

[26] Brun C (2022). Resistance to glycation in the zebra finch: mass spectrometry-based analysis and its perspectives for evolutionary studies of aging. Exp. Gerontol..

[27] Landon C, Thouzeau C, Labbe H, Bulet P, Vovelle F (2004). Solution structure of spheniscin, a beta-defensin from the penguin stomach. J. Biol. Chem..

[28] Bertile F (2016). The safety limits of an extended fast: lessons from a non-model organism. Sci. Rep..

[29] Medini D (2008). Microbiology in the post-genomic era. Nat. Rev. Microbiol..

[30] Ekblom R, Galindo J (2011). Applications of next generation sequencing in molecular ecology of non-model organisms. Heredity.

[31] Chen L (2014). Advances in genome editing technology and its promising application in evolutionary and ecological studies. Gigascience.

[32] Doudna JA, Charpentier E (2014). The new frontier of genome engineering with CRISPR-Cas9. Science.

[33] Salzberg SL (2019). Next-generation genome annotation: we still struggle to get it right. Genome Biol..

[34] Joyce AR, Palsson BO (2006). The model organism as a system: integrating ‘omics’ data sets. Nat. Rev. Mol. Cell Biol..

[35] Vistain LF, Tay S (2021). Single-cell proteomics. Trends Biochem. Sci..

[36] Schoof EM (2021). Quantitative single-cell proteomics as a tool to characterize cellular hierarchies. Nat. Commmun..

[37] Perkel JM (2021). Single-cell proteomics takes centre stage. Nature.

[38] Leipert J (2023). Digital microfluidics and magnetic bead-based intact proteoform elution for quantitative top-down nanoproteomics of single C. elegans nematodes. Angew. Chem. Int. Ed. Engl..

[39] Armengaud J (2023). Metaproteomics to understand how microbiota function: the crystal ball predicts a promising future. Environ. Microbiol.

[40] Yang Y (2020). Genomic, transcriptomic, and proteomic insigths into the symbiosis of deep-sea tubeworm holobionts. ISME J..

[41] Cassidy L, Tholey A (2014). Model organism proteomics as a tool for the study of host-microbiome interactions. Proteom. Clin. Appl..

[42] Heck M, Neely BA (2020). Proteomics in non-model organisms: a new analytical frontier. J. Proteome Res..

[43] Muth T, Renard BY (2018). Evaluating de novo sequencing in proteomics: already an accurate alternative to database-driven peptide identification?. Brief. Bioinform..

[44] Wang Z, Gerstein M, Snyder M (2009). RNA-seq: a revolutionary tool for transciptomics. Nat. Rev. Genet..

[45] Prata, J. C., Ribeiro, A. I. & Rocha-Santos, T. in *One Health* (eds J. C. Prata, A. I. Ribeiro, & T. Rocha-Santos) Ch. 1, 1–31 (Academic Press, 2022).

[46] Leonelli S, Ankeny RA (2012). Re-thinking organisms: the impact of databases on model organism biology. Stud. Hist. Philos. Biol. Biomed. Sci..

[47] Subramanian I, Verma S, Kumar S, Jere A, Anamika K (2020). Multi-omics data integration, interpretation, and its application. Bioinform. Biol. Insigths.

[48] Mani DR (2022). Cancer proteogenomics: current impact and future prospects. Nat. Rev. Cancer.

[49] Smith LM, Kelleher NL (2018). Proteoforms as the next proteomics currency. Science.

[50] Cassidy L, Kaulich PT, Tholey A (2023). Proteoforms expand the world of microproteins and short open reading frame-encoded peptides. iScience.

[51] Marchione AD, Thompson Z, Kathrein KL (2021). DNA methylation and histone modifications are essential for regulation of stem cell formation and differentiation in zebrafish development. Brief. Funct. Genom..

[52] Su T, Hollas MAR, Fellers RT, Kelleher NL (2023). Identification of splice variants and isoforms in transcriptomics and proteomics. Annu. Rev. Biomed. Data Sci..

